# Can preoperative liver MRI with gadoxetic acid help reduce open-close laparotomies for curative intent pancreatic cancer surgery?

**DOI:** 10.1186/s40644-021-00416-4

**Published:** 2021-06-30

**Authors:** Kartik S. Jhaveri, Ali Babaei Jandaghi, Seng Thipphavong, Osvaldo Espin-Garcia, Anna Dodd, Shawn Hutchinson, Trevor W. Reichman, Carol-Anne Moulton, Ian D. McGilvary, Steven Gallinger

**Affiliations:** 1 Joint Department of Medical Imaging, University Health Network, Mount Sinai Hospital and Women’s College Hospital, University of Toronto, 610 University Ave, 3-957, Toronto, ON M5G 2M9 Canada; 2 Joint Department of Medical Imaging, University Health Network, Mount Sinai Hospital and Women’s College Hospital, Toronto, ON M5G 1X6 Canada; 3 Department of Biostatistics, Princess Margaret Cancer Centre, University Health Network, Toronto, ON M5G 2C1 Canada; 4 Division of Biostatistics, Dalla Lana School of Public Health, University of Toronto, Toronto, Canada; 5 Wallace McCain Centre for Pancreatic Cancer, Princess Margaret Hospital Cancer Centre, Toronto, ON M5G 2C1 Canada; 6 Department of Surgery, University of Toronto; Hepatobiliary & Pancreatic Surgical Oncology, University Health Network, Toronto, ON M5G 2C4 Canada; 7 Department of Surgery, Hepatobiliary & Pancreatic Surgical Oncology, University Health Network, University of Toronto, Toronto, ON M5G 2N2 Canada

**Keywords:** Pancreatic adenocarcinoma, Gadoxetic acid-enhanced MRI, Liver metastasis, Whipple procedure, CT scan

## Abstract

**Objectives:**

To evaluate gadoxetic acid-enhanced liver MRI (EOB-MRI) versus contrast-enhanced computed tomography (CECT) for preoperative detection of liver metastasis (LM) and reduction of open-close laparotomies for pancreatic ductal adenocarcinoma (PDAC).

**Methods:**

Sixty-six patients with PDAC had undergone preoperative EOB-MRI and CECT. LM detection by EOB-MRI and CECT and their impact on surgical planning, open-close laparotomies were compared by clinical and radiology reports and retrospective analysis of imaging by two blinded independent readers. Histopathology or imaging follow-up was the reference standard. Statistical analysis was performed at patient and lesion levels with two-sided McNemar tests.

**Results:**

EOB-MRI showed higher sensitivity versus CECT (71.7% [62.1-80.0] vs. 34% [25.0-43.8]; *p* = 0.009), comparable specificity (98.6%, [96.9-99.5] vs. 100%, [99.1-100], and higher AUROC (85.1%, [80.4-89.9] vs. 66.9%, [60.9-73.1]) for LM detection. An incremental 7.6% of patients were excluded from surgery with a potential reduction of up to 13.6% in futile open-close laparotomies due to LM detected on EOB-MRI only.

**Conclusions:**

Preoperative EOB-MRI has superior diagnostic performance in detecting LM from PDAC. This better informs surgical eligibility with potential reduction of futile open-close laparotomies from attempted curative intent pancreatic cancer surgery.

**Supplementary Information:**

The online version contains supplementary material available at 10.1186/s40644-021-00416-4.

## Synopsis for table of contents

- Gadoxetic acid-enhanced liver MRI has superior diagnostic performance to CT for detection of liver metastases from pancreatic carcinoma.
- Gadoxetic acid-enhanced liver MRI provides incremental value for preoperative staging and surgical resection eligibility of pancreatic carcinoma.
- Inclusion of gadoxetic acid-enhanced liver MRI can reduce the incidence of failed curative intent pancreatic cancer surgeries (open-close laparotomies).

## Introduction

Pancreatic ductal adenocarcinoma (PDAC) has a poor prognosis with an overall 5-year survival rate of approximately 8% (range 3-32% depending on the stage) [1]. Generally, surgical resection is the only potentially curative treatment for pancreatic ductal adenocarcinoma (PDAC) [2] and unlike colorectal cancer, preoperative detection of liver metastasis (LM) precludes curative surgery. Since PDAC often presents late, only approximately 20% of patients are resectable at presentation, and in many, the disease has already metastasized [3, 4]. Contrast-enhanced computed tomography (CECT) is the most commonly used imaging modality for staging pancreatic cancer with its ability to detect local extension and distant metastasis [5, 6]. Unexpected adverse findings, in particular LM, have been reported intraoperatively in 8-26% with potentially resectable pancreatic cancer on CECT [7–9], resulting in aborted surgeries [10]. Thus, it is of great relevance to detect LM on preoperative imaging to avoid futile (open-close) laparotomies and resultant associated postoperative morbidity/mortality and added hospitalization costs [11].

Gadoxetic acid (Gd-EOB-DTPA) is a hepatobiliary MRI contrast agent [Eovist (US) or Primovist (Canada, Europe), Bayer HealthCare, NJ, USA] with both extracellular and liver-specific properties. Its uptake by hepatocytes and biliary excretion enhances the contrast between the liver parenchyma and hepatic lesions, thereby improving liver lesion detection [12]. Some studies have reported the incremental value of Gadoxetic acid-enhanced liver MR (EOB-MRI) over CECT for detection of LM, particularly in colorectal cancer [13, 14]. It has recently been shown that even though EOB-MRI had similar performance to extracellular-enhanced MR imaging in detecting PDAC, it had greater sensitivity in detecting LM [15]. Although a few studies have evaluated the added value of EOB-MRI over CECT in identifying LM from pancreatic cancer [10, 11], specifically PDAC [16], assessment of direct impact on surgical management of PDAC is not well reported.

This study aimed to compare the diagnostic performance of EOB-MRI versus CECT in the preoperative detection of LM in patients being considered for curative-intent PDAC surgery and its impact on surgical planning and reduction of futile (open-close) laparotomies.

## Materials and methods

### Patients

**Fig. 1:**
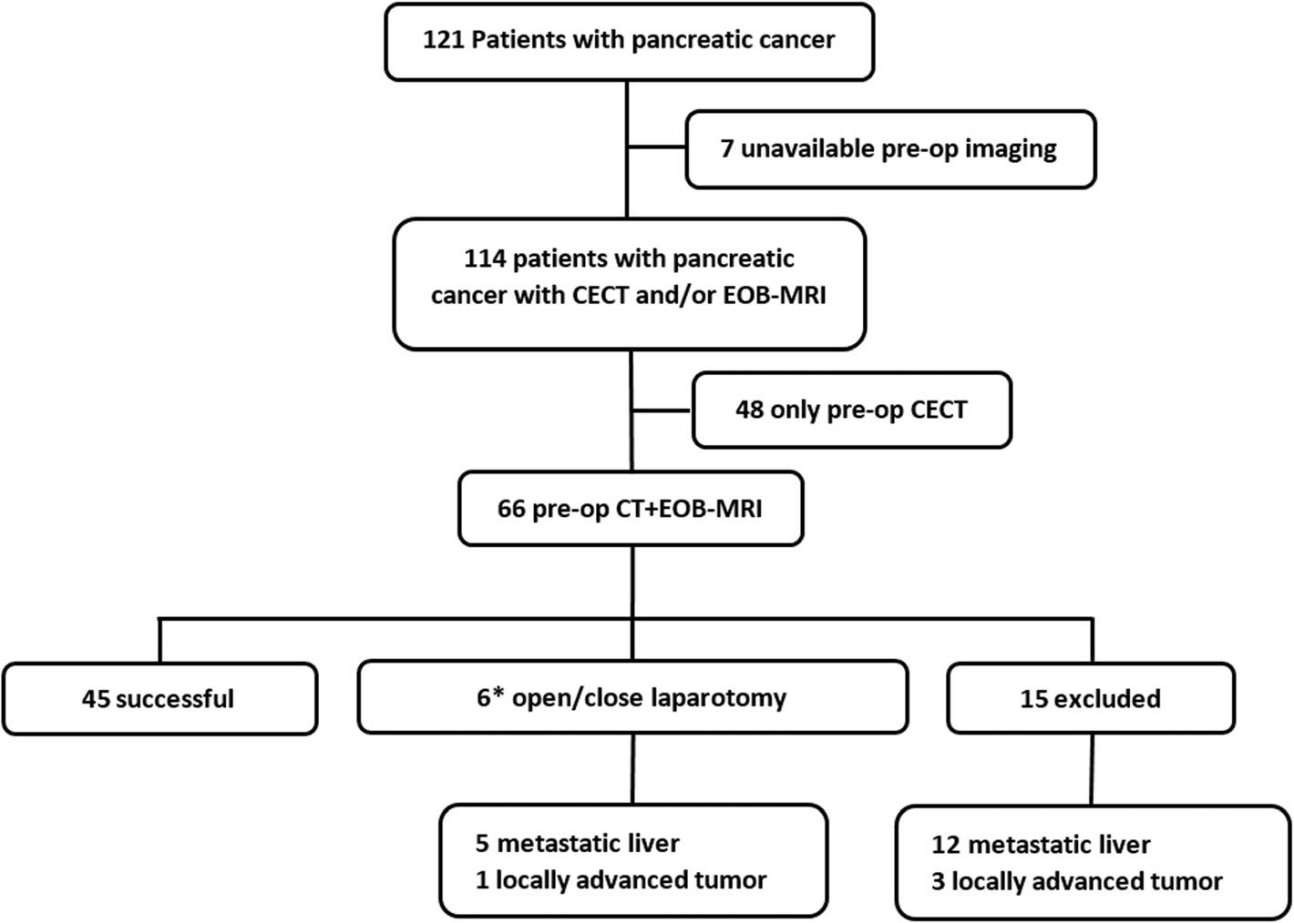
Patient enrollment flowchart and surgical outcomes of patients with pancreatic cancer. *Although EOB-MRI correctly diagnosed two patients with liver metastases, the clinical decision was to proceed to laparotomy and perform excisional biopsy, which confirmed liver metastasis. Therefore, the number of open-close laparotomies could have actually been reduced to 4 in this category

This was a single-institution HIPAA compliant, retrospective study with research ethics board approval and patient consent waiver. All consecutive patients with PDAC who had undergone preoperative CECT and EOB-MRI as per institutional standard of care from January 2018 to October 2019 were included. Those with incomplete or unavailable clinical, imaging or surgical data were excluded. Patient demographic characteristics (age and sex), pancreatic tumor location, tumor histopathology, carbohydrate antigen 19-9 [CA19-9], intraoperative observations, surveillance imaging and clinical outcomes were collected from the institutional electronic patient record (EPR) system. One hundred twenty-one patients with PDAC were referred to our institution for pancreatic cancer management during the study period. Seven patients were excluded due to lack of preoperative imaging and forty-eight patients because of unavailability of EOB-MRI. The final study cohort comprised of 66 patients with PDAC (37 males and 29 females with a mean age of 64.8 ± 10.4 years, range 35-85 years) who had undergone both preoperative CECT and EOB-MRI. (Fig. 1) The patient demographics and clinicopathologic characteristics are detailed in Table 1. The absolute median time intervals between CECT and EOB-MRI (12 days, IQR [5-25], range 0-119 days) and between the latest preoperative imaging and surgery (10 days, IQR [7-23.5], range 2-101 days) were less than 2 weeks. Study data were managed using REDCap (Vanderbilt University Medical Center, TN, USA) electronic data capture tools hosted at our institution [17]. The original imaging report observations as well as secondary reader interpretations of CECT and EOB-MRI were compared to the reference standard for LM diagnosis.

**Table 1:** Patient Demographic and Clinicopathologic Characteristics

All patients		66
**Characteristic**		
**Sex**	F	29 (44)
	M	37 (56)
**Median Age [Range], years**		66 [36-85]
**Primary surgical procedure**	Whipple	34 (51.6)
	Total Pancreatectomy	2 (3.0)
	Distal Pancreatectomy	9 (13.6)
	Open/close Surgery	6 (9.1)
	Excluded Surgery	15 (22.7)
**NAC**		13 (19.7)
**Tumor location**	Head	49 (74.3)
	Neck	3 (4.5)
	Body	8 (12.1)
	Tail	6 (9.1)
**Resection margin status**	R0	38 (57.6)
	R1	7 (10.6)
	NA (aborted/excluded Surgery)	21 (31.8)
**Pathologic grades**	Well-differentiated (G1)	2 (3.0)
	Moderately-differentiated (G2)	42 (63.7)
	Poorly-differentiated (G3)	8 (12.1)
	Cannot be assessed (Gx)	2 (3.0)
	Unknown (FNA, CNB of metastasis)	12 (18.2)
**Lymphovascular invasion**	Yes	24 (36.4)
	No	19 (28.8)
	Indeterminate	2 (3.0)
	Unknown (aborted/excluded Surgery)	21 (31.8)
**Perineural invasion**	Yes	43 (65.2)
	No	1 (1.5)
	Indeterminate	1 (1.5)
	Unknown (aborted/excluded Surgery)	21 (31.8)
**Pathologic T stage**	1	3 (4.5)
	2	29 (44)
	3	11 (16.7)
	4	1 (1.5)
	Cannot be assessed (pTx)	1 (1.5)
	Unknown (aborted/excluded surgery)	21 (31.8)
**Pathologic N stage**	0 (no regional LN metastasis)	13 (19.7)
	1 (regional LN metastasis 1-3)	12 (18.2)
	2 (regional LN ≥ 4)	20 (30.3)
	Unknown (aborted/excluded Surgery)	21 (31.8)
**Pathologic stage**	I	9 (13.6)
	IIA	4 (6.1)
	IIB	32 (48.5)
	III	1 (1.5)
	IV	1 (1.5)
	Unknown (aborted/excluded Surgery)	19 (28.8)
**Preoperative serum CA19-9**	Normal (≤ 34 U/mL)	10 (15.1)
	Abnormal (> 34 U/mL)	50 (75.8)
	Not available	6 (9.1)

Data in parentheses are numbers used to calculate the percentages. *CECT* Contrast-enhanced computed tomography, *F* Female, *M* Male, *NAC* Neoadjuvant chemotherapy, *NA* Not applicable, *FNA* Fine needle aspiration, *CNB* Core needle biopsy, *LN* Lymph node

### Imaging techniques

CECT was performed on 64-MDCT scanners (Aquilion 64, Toshiba CA, USA) using arterial and venous phases. Scanner parameters were as follows: axial slice thickness, 2 mm; interval, 2 mm; detector configuration, 64 × 0.5 (32 mm); tube voltage, 120 kV; automated tube current of 100–500 mA, helical pitch, 53; pitch factor, 0.828; tube rotation time of 0.5 s; and slice thickness for reconstructed coronal and sagittal planes, 3 mm. The craniocaudal coverage for arterial phase was from top of the liver to below the duodenum, including the entire liver, and for venous phase was from top of the diaphragm to the iliac crest. A dose of 2 ml/kg to a maximum of 150 mL non-ionic contrast agent (Ultravist 370, GE Healthcare) was administered intravenously, through a 22-gauge intravenous catheter inserted into a forearm vein, with a power injector [Medrad® Stellant® Dual Head] at a rate of 3 mL/s. Arterial and venous phases were performed on abdominal aorta at 100 HU plus 25 s and 60 s, respectively. Multiplanar reformatted images in coronal and sagittal planes were reconstructed for all phases with a slice thickness of 3 mm.

EOB-MRI was performed on 1.5 T (Magnetom Avanto; Siemens Healthcare, Erlangen, Germany) or 3 T MR system (Magnetom Verio with Tim system; Siemens Health care, Erlangen, Germany) with multichannel phased array coils (16 or 32 channels) using a standard liver EOB-MRI protocol (Table 2).

**Table 2:** Protocol for gadoxetic acid-enhanced liver MRI (EOB-MRI)

Image sequence	Field strengths	TR (ms)	TE (ms)	FA (D^**°**^)	FOV (mm)	ST (mm)	Voxel size (mm)	Fat suppression	Respiratory control
**Axial T2 HASTE**	**1.5 T**	1200	180	160	400	5	1.3 × 1.3 × 5.0	SPAIR	Breath-hold
	**3 T**	1200	181	146	380	5	1.2 × 1.2 × 5.0		
**Coronal T2 HASTE**	**1.5 T**	1200	180	160	400	4	1.3 × 1.3 × 4.0	None	Breath-hold
	**3 T**	1600	180	160	380	4	1.2 × 1.2 × 4.0		
**Axial T1 VIBE opp phase**	**1.5 T**	178	2.22-4.60	70	380	4	0.7 × 0.7 × 4.0	None	Breath-hold
	**3 T**	150	1.23-2.46	70	380	4	0.7 × 0.7 × 4.0		
**ep2d diff b100,600**	**1.5 T**	5600	63.0	–	440	5	1.1 × 1.1 × 5.0	SPAIR	Off
	**3 T**	6100	68.0	–	400	5	1.0 × 1.0 × 5.0		
**Axial T1 VIBE**	**1.5 T**	4.07	1.92	10	420	3	1.3 × 1.3 × 3.0	SPAIR	Breath-hold
	**3 T**	3.60	1.75	9	380	3	1.2 × 1.2 × 3.0		
**Post-contrast imaging**^**a**^									
**Axial T1 VIBE** **(late arterial phase)**	**1.5 T**	4.07	1.92	10	420	3	1.3 × 1.3 × 3.0	SPAIR	Breath-hold
	**3 T**	3.60	1.75	9	380	3	1.2 × 1.2 × 3.0	SPAIR	Breath-hold
**Axial T1 VIBE** **(portal venous phase)**	**1.5 T**	4.07	1.92	10	420	3	1.3 × 1.3 × 3.0	SPAIR	Breath-hold
	**3 T**	3.60	1.75	9	380	3	1.2 × 1.2 × 3.0	SPAIR	Breath-hold
**Axial T1 VIBE (transitional phase)**	**1.5 T**	4.07	1.92	10	420	3	1.3 × 1.3 × 3.0	SPAIR	Breath-hold
	**3 T**	3.60	1.75	9	380	3	1.2 × 1.2 × 3.0	SPAIR	Breath-hold
**Axial T1 VIBE 20 min (hepatobiliary phase)**	**1.5 T**	4.97	2.01	25	400	3	1.3 × 1.3 × 3.0	SPAIR	Breath-hold
	**3 T**	4.29	1.82	25	360	3	1.1 × 1.1 × 3.0		
**Coronal T1 VIBE 20 min**	**1.5 T**	4.29	2.01	25	400	3	1.3 × 1.3 × 3.0	SPAIR	Breath-hold
	**3 T**	4.42	1.77	25	400	3	1.3 × 1.3 × 3.0		
**Axial T1 CAIPI-VIBE 20 min (hepatobiliary phase)**	**1.5 T**	6.63	TE1 = 2.36 TE2 = 4.77	9	360	1.5	1.1 × 1.1 × 1.5	None	Breath-hold
	**3 T**	4.02	TE1 = 1.32 TE2 = 2.55	9	360	1.5	1.1 × 1.1 × 1.5		

^a^Patients received Gadoxetic acid (Primovist or Eovist, Bayer AG, Germany) intravenously through a 22-gauge intravenous catheter inserted into a forearm vein with an MR-compatible power injector (Medrad® Spectris Solaris® EP MR Injection system, Bayer Healthcare, Whippany, USA) at a rate of 1 mL/s (0.025 mmol/kg body weight) followed by a 10-mL normal saline chaser at the same rate

*FA* Flip angle, *FOV* Field of view, *SPAIR* Spectral Attenuated Inversion Recovery, *ST* Section thickness, *TE* Echo time, *TR* Repetition time

### Patient and image analysis

We analyzed the formal reports of the preoperative EOB-MRI and CECT from our Radiology Information System (RIS) for the diagnosis of LM verified as per the reference standard. The number, location, size, and final radiologist diagnosis of all reported liver lesions were recorded from the reports according to a 3-point confidence scale scoring system (benign, indeterminate, and malignant). The final imaging diagnosis (malignant, benign, or indeterminate) per patient was also categorized according to the most concerning liver lesion reported on CECT and EOB-MRI. The actual impact of EOB-MRI during preoperative surgical planning was determined by the number of patients excluded from curative surgery because of LM detected incrementally on EOB-MRI over CECT as documented in clinical charts.

Additionally, two subspecialized abdominal radiologists each with 20 and 10 years of experience, independently and in blinded fashion retrospectively evaluated the anonymized EOB-MRI and CECT examinations with a gap of more than 4 weeks between the two modalities to minimize recall bias probability. All images were reviewed on the imaging workstations (Coral PACS, Version: 2.5.0.0, JDMI Informatics, University Health Network, Toronto, Canada). All visible liver lesion locations based on Couinaud segmental anatomy were recorded and measured on the axial T1-weighted post-contrast hepatobiliary phase of EOB-MRI and portal venous phase of CECT. The lesion imaging characteristics and final imaging diagnosis were again recorded, utilizing the same 3-point confidence scale scoring system for both modalities using standard diagnostic imaging criteria used in clinical practice (Additional file 1) [18, 19]. The reader diagnosis for LM was verified against the reference standard.

The potential impact of EOB-MRI on preoperative surgical planning based on the detection of LM on CECT versus EOB-MRI by the two readers was compared. Readers determined simulated eligibility for curative pancreatic cancer surgery based on LM detection. The differences in patient eligibility between CECT and EOB-MRI based on LM, which would have resulted in potential futile (open-close) surgery attempts, was calculated for each reader. Inter-reader agreements for LM detection and simulated decision-making process were also evaluated.

### Reference standard

Histopathology, intraoperative observations, and/or interval follow-up were considered as the reference standard for the diagnosis of LM. Suspected LM were evaluated by either pre−/intra-operative biopsy or interval follow-up imaging. In the presence of multiple suspected LM with similar imaging characteristics, a preoperative biopsy was performed only from one of the lesions, and the remainder were evaluated on follow-up imaging for change in lesion size/morphology. On follow-up imaging, a suspicious liver lesion was considered as metastasis if it showed ≥20% increase in axial diameter within a 3-month interval.

### Statistical analysis

A patient-level analysis of preoperative EOB-MRI and CECT original reports to measure the diagnostic performance of the modalities in detection of LM and the actual impact of EOB-MRI on surgical planning during the prospective clinical decision-making process was performed. Additionally, patient-level and lesion-level analyses to evaluate the diagnostic performance of CECT and EOB-MRI imaging in detection of LM and the impact of EOB-MRI versus CECT on simulated surgical eligibility and differences in potential futile (open-close) laparotomies, for which McNemar tests were carried out. Imaging scores were summarized for each reader by modality (EOB-MRI, CECT) on a 3-point scale (metastasis, indeterminate, benign). Inter-observer agreement in scoring hepatic lesions based on a binary classification treating indeterminate as benign was evaluated by reporting the concordance rate (%) of diagnosis between the readers for both EOB-MRI and CECT. To calculate the sensitivity and corresponding 95% confidence intervals (CIs) of EOB-MRI and CECT in detecting LM in correlation with the reference standard, we used generalized estimating equations (GEE) to account for repeated measurements within a patient [20]. The CIs were computed under the Clopper–Pearson exact method. At the patient level, the potential impact of EOB-MRI on surgical planning was summarized for each reader.

All tests were two-sided, and a *P*-value < 0.05 was considered an indicator of a statistically significant difference. Statistical analyses were performed using R version 3.5.0 [21].

## Results

Curative-intent pancreatic cancer surgery was excluded in 15 patients [LM in 12, and vascular issues (occlusion or encasement by the primary tumor) in 3 patients]. Amongst 51 patients with attempted curative-intent surgery, 45 had successful surgeries, while 6 had open-close laparotomies. The reasons for open-close laparotomy were surface LM (*n* = 3), intraparenchymal LM (*n* = 2), and unexpected common hepatic arterial tumor encasement (*n* = 1). (Fig. 1) The mean size of LM discovered during surgery was 6.4 mm (2-10 mm). The intraparenchymal LM were retrospectively detectable only on MRI. Approximately 17% (11/66) of patients had received preoperative neoadjuvant chemotherapy for downstaging of vascular involvement before surgery. None of these patients had LM before starting the neoadjuvant chemotherapy. The mean time interval between neoadjuvant chemotherapy and preoperative imaging was 3.3 ± 1.3 months.

**Fig. 2:**
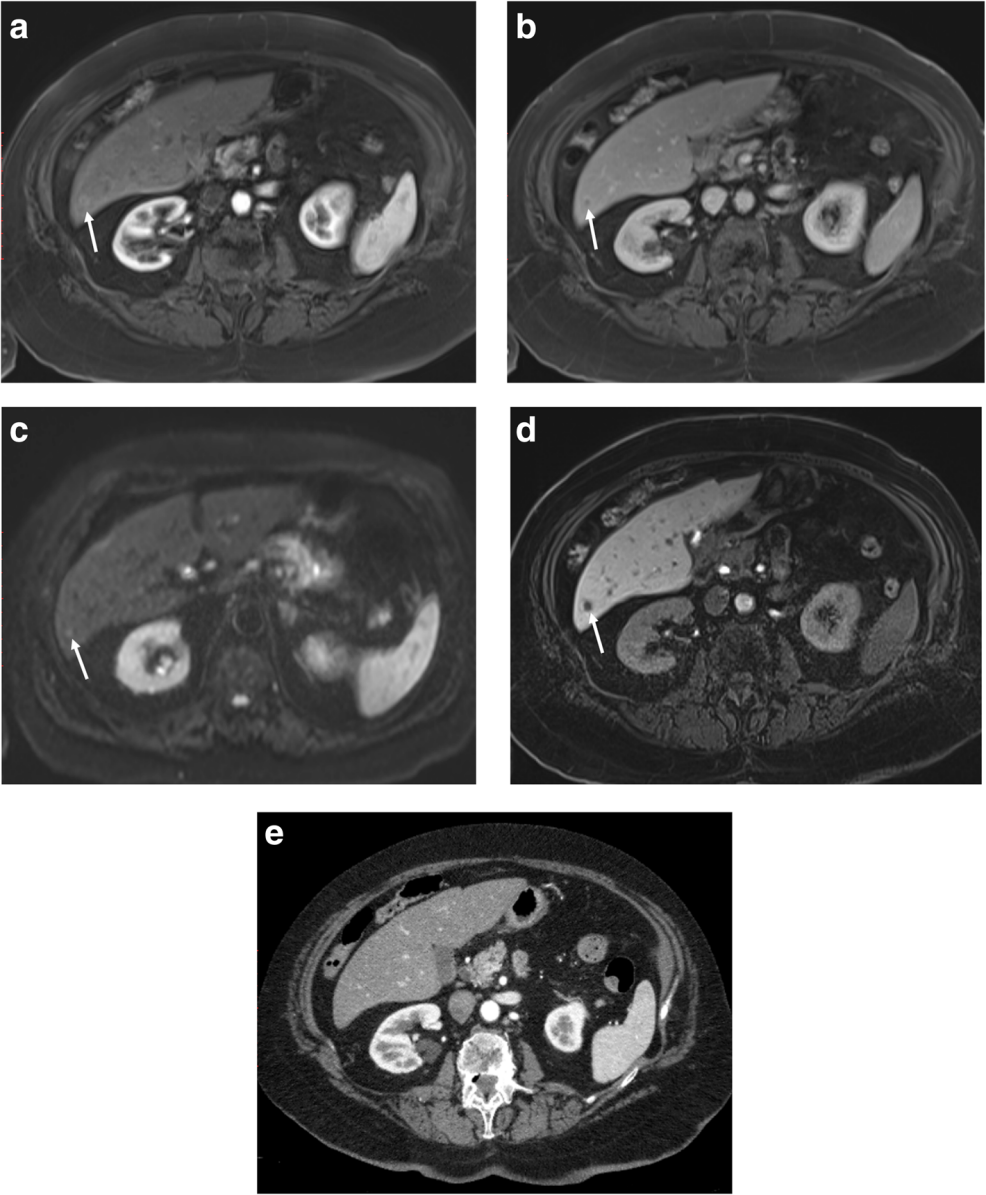
A 78-year-old female with otherwise resectable pancreatic cancer. A small liver metastasis (arrows) was diagnosed in segment 6 on EOB-MRI (**a**-**d**), but was invisible on CECT (**e**). While the lesion (arrows) shows faint rim enhancement in arterial (**a**) and portal (**b**) phases, and diffusion restriction in DWI (**c**), it is clearly detectable on hepatobiliary phase (**d**). This impacted the surgical plan (compared to CECT alone) with cancellation of curative-intent surgery and potential avoidance of a futile laparotomy

In the patient-level analysis of the formal imaging reports, the sensitivity of EOB-MRI for LM was significantly higher than CECT {70.6% (12/17) [46.6-86.8], vs 29.4% (5/17) [13.9-51.8], *p* = 0.002}. The specificity, positive and negative predictive values (PPV, NPV), and accuracy were 98% [87.7-99.7], 92.3% [55.2-99.1], 90.6% [74.9-96.0], and 90.9% [81.2-95.9] versus 100% [92.7-100], 100% [47.8-100], 80.3% [59.4-88.4], and 81.8% [70.7-89.4] for EOB-MRI and CECT, respectively. Although two patients were diagnosed with intraparenchymal LM on preoperative EOB-MRI, the clinical decision was to attempt surgery and perform excisional biopsies, which confirmed metastasis in both patients leading to open-close laparotomies (Fig. 2). Excluding these two patients from surgical exploration based on EOB-MRI would have further lowered the actual open-close laparotomy rate to 6.1% (4/66) from 9.1% (6/66). Among patients excluded from surgery due to LM (12/66), 10/12 patients with LM were identified by EOB-MRI while CECT identified only 5/12 patients. Thus, an incremental 7.6% (5/66) of patients were excluded from surgery due to LM detected only on EOB-MRI and CECT detected only 50% of the patients with LM compared to EOB-MRI. Two patients with LM were reported as indeterminate on both EOB-MRI and CECT. One of these patients had pancreatic tumor invading to portal vein confluence, and surgery was excluded on those grounds with follow-up imaging also indirectly confirming LM. In the other patient, percutaneous biopsy of an indeterminate liver lesion confirmed LM, thereby excluding surgery.

**Fig. 3:**
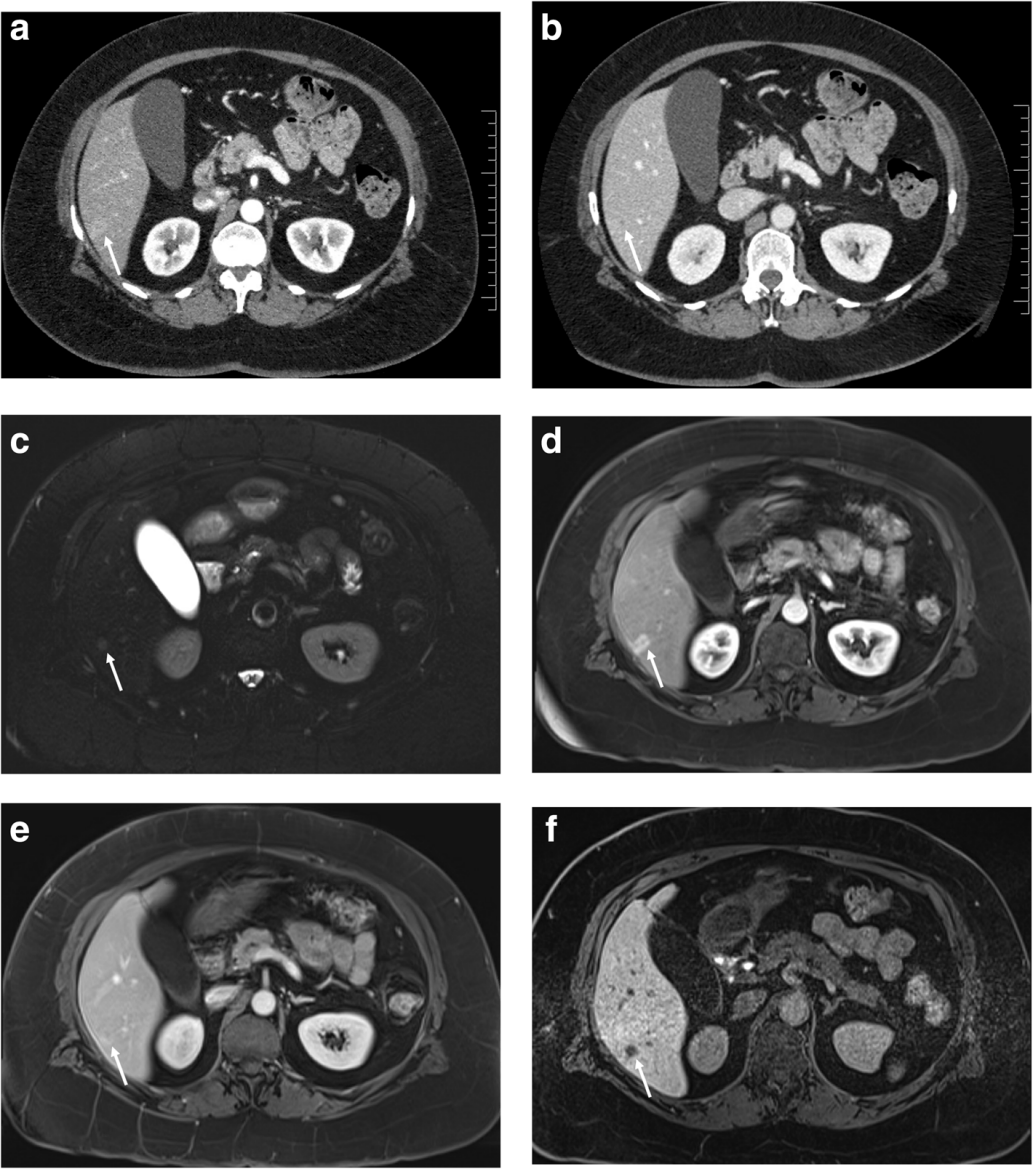
A 55-year-old female with pancreatic cancer and indeterminate lesion on CECT but diagnosis of liver metastasis on EOB-MRI (arrows). There is a vascular blush in segment 6 on arterial phase of CT scan (**a**), measuring 9 mm. On the portal phase of CT scan (**b**), a subtle, indeterminate low-attenuation lesion is evident. On EOB-MRI, a mildly T2 hyperintense nodule (**c**) demonstrates peripheral enhancement with central hypoenhancement in arterial (**d**) and portal (**e**) phases and marked hypointensity on the hepatobiliary phase (**f**). The patient was excluded from surgery, and the percutaneous focal liver lesion biopsy confirmed metastatic adenocarcinoma

In the lesion-level analysis of retrospectively performed blinded independent readers’ observations 261 liver lesions were recorded 208 benign lesions (mean size, 7 mm; range 2-59 mm) and 53 metastases (mean size, 8.4 mm; range: 2-19 mm) in 66 patients as per reference standard, including. Reader 1 and 2 reported 40 and 36 LM respectively on EOB-MRI compared to 18 LM by both readers on CECT. Mean reader results revealed that 81% (43/53) of LM were diagnosed on EOB-MRI and 38% (20/53) on CECT. The overall sensitivity of EOB-MRI was significantly greater than CECT for both readers (mean, 71.7% [62.1-80.0] vs. 34% [25.0-43.8]; *p* = 0.009; R1, 75.5% [61.7-86.2] vs. 34% [21.5-48.3]; *p* = 0.009; R2, 67.9% [53.7-80.1] vs. 34% [21.5-48.3]; *p* = 0.018). Also, EOB-MRI had comparable mean specificity (98.6%, [96.9-99.5] vs. 100%, [99.1-100], and higher AUROC (85.1%, [80.4-89.9] vs. 66.9%, [60.9-73.1]) for LM detection. Table 3 illustrates the diagnostic performance of CECT and EOB-MRI as per two blinded independent readers. EOB-MRI detected additional LM (size range, 2-19 mm; 25 and 20 metastatic lesions for R1 and R2, respectively) compared to CECT. The number of small additional metastasis (≤ 1.0 cm) detected on EOB-MRI was 18 for reader 1 and 14 for reader 2. The number of LM ≤1.0 cm detected on EOB-MRI was significantly higher for both readers [R1, 60.4% (32/53) vs. 37.7% (20/53), *p* = 0.009; R2, 56.6% (30/53) vs. 35.8% (19/53), *p* = 0.008]. Moreover, the number of indeterminate lesions was lower on EOB-MRI compared to CECT for both readers, and this difference was statistically significant for one of the readers (R1: 6.1% (16/261) vs. 13.0% (34/261), *p* = 0.039; R2: 7.7% (20/261) vs. 14.9% (39/261), *p* = 0.081). Amongst 10 LM characterized as indeterminate on CECT by both readers, 80 and 50% of those lesions were accurately diagnosed on EOB-MRI as metastases by readers 1 and 2, respectively. (Fig. 3) The inter-reader agreement for diagnosis of LM was high on EOB-MRI [κ = 0.76 (95% CIs 0.594-0.926)], while it was excellent on CECT [κ = 0.855 (95% CIs 0.663-1.047)].

**Table 3:** Diagnostic performance of EOB-MRI and CECT for detection of liver metastasis at lesion level

	EOB-MRI	CECT
**Sensitivity**		
Reader 1	75.5 (40/53) [61.7-86.2]	34 (18/53) [21.5-48.3]
Reader 2	67.9 (36/53) [53.7-80.1]	34 (18/53) [21.5-48.3]
Mean	71.7 (76/106) [62.1-80]	34 (36/106) [25-43.8]
**Specificity**		
Reader 1	98.1 (204/208) [95.1-99.5]	100 (208/208) [98.2-100]
Reader 2	99 (206/208) [96.6-99.9]	100 (208/208) [98.2-100]
Mean	98.6 (410/416) [96.9-99.5]	100 (416/416) [99.1-100]
**PPV**		
Reader 1	90.9 (40/44) [78.3-97.5]	100 (18/18) [81.5-100]
Reader 2	94.7 (36/38) [82.3-99.4]	100 (18/18) [81.5-100]
Mean	92.7 (76/82) [84.8-97.3]	100 (36/36) [90.3-100]
**NPV**		
Reader 1	94 (204/217) [90-96.8]	85.6 (208/243) [80.5-89.8]
Reader 2	92.4 (206/223) [88.1-95.5]	85.6 (208/243) [80.5-89.8]
Mean	93.2 (410/440) [90.4-95.4]	85.6 (416/486) [82.2-88.6]
**Accuracy**		
Reader 1	93.5 (244/261) [89.8-96.2]	86.6 (226/261) [81.8-90.5]
Reader 2	92.7 (242/261) [88.9-95.6]	86.6 (226/261) [81.8-90.5]
Mean	93.1 (486/522) [90.6-95.1]	86.6 (452/522) [83.4-89.4]

Data in parentheses are numbers used to calculate the percentages, the values in brackets are confidence intervals. *EOB-MRI* Gadoxetic acid-enhanced MRI, *CECT* Contrast-enhanced computed tomography, *PPV* Positive predictive value, *NPV* Negative predictive value

In a patient-level analysis of readers’ observations by considering only the presence or absence of LM, the sensitivity of EOB-MRI for detecting metastatic liver disease was again superior to that of CECT for both readers (R1, 75.4% [49.7-90.5] vs 35.3% [17.9- 57.7], *p* = 0.001; R2, 71.7% [45.1-88.6] vs 41.2% [19.6-66.8], *p* = 0.006). In addition, for both readers, the incidence of indeterminate diagnosis (score 3) was lower on EOB-MRI; and this difference between the two modalities was significant for reader 1 (R1, 13.6% [7.3-24.2] vs. 30.3% [20.5-42.4], *p* = 0.028; R2, 12.1% [6.2-22.4] vs. 21.2% [13-32.7]), *p* = 0.159). Table 4 demonstrates the potential change in surgical plan and reduction of futile open-close laparotomy rates based on each reader’s interpretation of EOB-MRI and CECT for LM. A potential 13.6% (R1, *p* = 0.008) and 10.6% (R2, *p* = 0.023) decrease in futile (open-close) laparotomies is realized when surgical planning included EOB-MRI instead of CECT alone. The inter-reader agreement for this simulated decision-making process was substantial for both CECT and EOB-MRI [κ = 0.744 (95% CIs 0.468-1.02) for CECT and κ = 0.691 (95% CIs 0.478-0.904) for EOB-MRI].

**Table 4:** Potential change in surgical plan due to higher yield of EOB-MRI in liver metastasis detection

	Ineligible for surgery		
	CECT	EOB-MRI	Potential reduction of futile (open-close) laparotomy
**Reader 1**	9.1% (6/66)	22.7% (15/66)	13.6% (9/66)
**Reader 2**	10.6% (7/66)	21.2% (14/66)	10.6% (7/66)

Data in parentheses are numbers used to calculate the percentages. *EOB-MRI* Gadoxetic acid-enhanced MRI, *CECT* Contrast-enhanced computed tomography

## Discussion

As per the current National Comprehensive Cancer Network (NCCN) guidelines (2021), the preferred imaging tool for pancreatic imaging is dual-phase pancreatic protocol CECT, whereas MRI is mostly used as a problem-solving tool (CT-indeterminate liver lesions, suspected tumor invisible on CECT, and contraindications for CECT, such as severe allergy to iodinated intravenous contrast material) [22]. However, there is a significant incidence of unnecessary laparotomy in up to 41% of patients with CT-defined resectable PDAC [5, 8, 9]. A meta-analysis assessing predicting resectability in patients with PDAC by CECT revealed a summary positive predictive value (sPPV) of 81%, indicating that 19% undergo surgical exploration only [5]. Considering the burden of cost and morbidity from futile surgery and the delay in the initiation of alternative treatments like chemotherapy, it is crucial to accurately select candidates for curative-intent surgery thereby decreasing the incidence of unnecessary laparotomies. Like MRI with extracellular contrast, MRI with liver-specific contrast could miss LM on dynamic arterial and portal phases. Yet, it can provide added value due to its hepatobiliary phase (HBP), in which both the hyper- and hypovascular metastases demonstrate hypointensity and increased conspicuity due to increased lesion-to-liver contrast. Generally, LM from PDAC are hypovascular and hypointense in arterial and portal-venous phases, and HBP could improve detection, thereby impacting surgical management. It has been shown that HBP can improve the detection of colorectal cancer LM, especially in the fatty liver or lesions smaller than 1 cm [13]. Our study demonstrates the beneficial impact of preoperative EOB-MRI on surgical planning due to its high diagnostic accuracy in LM detection with a potential reduction in the incidence of aborted curative-intent pancreatic cancer surgeries. At both patient and lesion levels, this study confirms superior sensitivity of EOB-MRI in depicting LM compared to that of CECT. Further, CECT was able to detect only half of the patients with LM compared to EOB-MRI. A few previous studies have reported the superior sensitivity of EOB-MRI in the detection of LM in pancreatic cancer [10, 11], specifically PDAC [16]. Motosugi et al. found that EOB-MRI was equivalent to CECT in depicting PDAC, and had a higher sensitivity for detecting LM [16]. Chew et al. reported sensitivity and specificity of 76.2 and 95.8% respectively for EOB-MRI in detecting LM in 69 patients with resectable pancreatic cancer on CECT, comparable with our mean reader results [11]. Ito et al. demonstrated that a significant predictor of LM was the identification of possible lesions on EOB-MRI, while other preoperative clinical factors, such as tumor markers and CECT findings were not predictive of LM [10]. Recently, a systematic review [23] reported that MRI was more sensitive than CT in detecting pancreatic cancer LM. This study supports our results; however, not all reviewed studies compared EOB-MRI with CECT in patients with PDAC while only two included studies performed MRI with a liver-specific agent in PDAC. Besides, this systematic review did not evaluate the impact of EOB-MRI versus CECT towards reduction of futile laparotomies. In our study, over one-tenth of patients with CT-defined resectable PDAC were incrementally detected to have LM on EOB-MRI rendering them unresectable. Mean reader results also revealed an incremental number of LM on EOB-MRI with apparent disease-free liver on CECT in a significant number of patients. Unlike CECT, EOB-MRI was able to correctly diagnose all patients with intraparenchymal LM, but similar to CECT, it failed to identify tiny liver surface metastases in a few patients leading to aborted surgery. These observations suggest that apart from small surface LM, EOB-MRI is highly reliable for detecting intraparenchymal LM in PDAC.

At a lesion level, our study showed that EOB-MRI had very high PPV and NPV for diagnosis of LM from PDAC. Likewise, at patient level, EOB-MRI had higher sensitivity and NPV than CECT for accurately identifying patients with LM. The high PPV of EOB-MRI for metastatic liver disease guides appropriate exclusion of curative-intent surgery in those patients, while the higher NPV compared to CECT can provide greater confidence towards proceeding with curative-intent surgery should there be no other contraindications. At lesion level, EOB-MRI was also significantly better than CECT in detection and characterization of small LM (≤1.0 cm). This result is also compatible with previous studies that evaluated the detection of LM on EOB-MRI [13, 24].

The incremental yield of EOB-MRI in detecting LM compared to CECT can reduce futile open-close laparotomies. Practically, in this study, surgery had been planned after the inclusion of both EOB-MRI and CECT findings. Therein EOB-MRI led to a two-fold exclusion of patients from attempted curative-intent PDAC surgery versus CECT due to detection of LM on EOB-MRI only. Additionally, the simulated rate of open-close surgery was significantly lower when the surgical plan was based on the detection of LM on EOB-MRI compared to CECT alone. This difference represents the potential incremental impact of EOB-MRI on clinical decision making and can reduce unnecessary open-close laparotomies. Additionally, reader interpretations of EOB-MRI demonstrated greater diagnostic confidence for lesion characterization with a lower incidence of indeterminate liver lesion diagnoses compared to CECT. Since conclusive preoperative imaging reports are, incontrovertibly, more helpful in making surgical decisions than those with indeterminate diagnosis, EOB-MRI further also helps in reducing diagnostic uncertainty regarding the presence or absence of LM during the preoperative clinical decision-making process.

Staging laparoscopy (SL) is not routinely utilized at our institution during the preoperative staging of PDAC. Although some NCCN member institutions routinely use laparoscopy before surgery or chemoradiation to rule out undetected metastases on imaging, there is a lack of consensus on the routine use of staging laparoscopy (SL) [25, 26]. Further, in most studies, the utility of SL was compared with preoperative CECT and not EOB-MRI. De Rosa et al. reviewed 24 studies assessing the utility of SL for CECT-defined resectable PDAC and demonstrated that the role of SL remains controversial. Although some of the advantages of SL include low rates of morbidity and mortality, and detection of peritoneal and liver surface metastases, it cannot adequately evaluate the posterior surface of liver or detect deeply located intraparenchymal LM with a false-negative rate of up to 9% having been reported [27]. In terms of cost-effectiveness, again, most studies have analyzed the cost-effectiveness of SL against the diagnostic performance of CECT and not EOB-MRI [28, 29]. In contrast, a retrospective indirect cost-effectiveness analysis of SL after MRI for pancreatic head carcinoma concluded that considering the paucity of missed metastases on MRI, the cost-effectiveness of SL is poor, and its yield is marginal. The NCCN panel suggests that SL can be considered for patients staged with resectable pancreatic cancer at increased risk for disseminated disease (markedly elevated CA 19-9, large primary tumors, large lymph nodes) and those with a borderline resectable disease before administration of neoadjuvant therapy [22]. In the future, it could be promising to assess if EOB-MRI can reduce the need for SL, consequently saving time, cost and potential morbidities.

Less than one-fifth of our patients had neoadjuvant chemotherapy, which may potentially increase the incidence of liver steatosis, and therefore decrease the sensitivity of CECT to detect LM. A previous study showed the superiority of EOB-MRI to CECT in the detection of small colorectal metastasis following chemotherapy, particularly in patients with hepatic steatosis [13]. They found a positive association between metastasis size of ≤1.0 cm detected only on EOB-MRI and the presence of hepatic steatosis. Our study did not find any significant increase in the presence of steatosis on chemical sift MRI versus CECT in those who had received chemotherapy. Therefore, we do not believe that neoadjuvant chemotherapy had significant influence on the results of our study.

Dual-energy CT (DECT), a relatively new imaging technology, allows characterization of tissue composition, such as calcium, iodine, and fat, and improves detection of pathologies by using two X-ray beams of different kVp energies [30, 31]. One of the stated benefits of DECT over conventional CT scan can be better characterization of small hypoattenuating liver lesions (cysts vs. LM) using iodine images. However, given limited availability and higher costs of DECT, it is not widely available for use in daily clinical practice. In the future, perhaps further studies directly comparing DECT with conventional CT scan or DECT with EOB-MRI might be valuable in preoperative surgical staging for patients with PDAC.

We must acknowledge some limitations of our study. First, this is a retrospective analysis with a relatively small patient cohort at a single site. However, all the study cohort patients underwent both EOB-MRI and CECT for direct head-to-head comparison of LM detection and impact on surgical decision-making process. In order to justify routine inclusion of EOB-MRI in all patients under consideration for curative pancreatic surgery, perhaps a prospective multi-center study with a larger cohort is required. Second, we did not specifically evaluate DWI diagnostic performance separately against the hepatobiliary phase of EOB-MRI. Our study aimed to perform a comprehensive evaluation of EOB-MRI rather than assessing portions of the MRI examination. A systematic meta-analysis assessing LM detection in patients with colorectal or non-colorectal cancer has shown lower sensitivity of DWI compared to EOB-MR imaging or the combination of both sequences in [32] with the latter having the highest sensitivity on a per-lesion basis. Finally, not all suspicious liver lesions were pathologically proven. If there were more than one suspicious liver lesion, tissue sampling would be performed first from the largest or the most accessible one, and further plans would be made accordingly. Disease progression on the subsequent follow-up imaging was used as a reference standard to confirm the metastatic nature of the unbiopsied lesions.

## Conclusions

EOB-MRI provides superior diagnostic performance compared to CECT in the preoperative detection of LM from PDAC. Moreover, this incremental yield of EOB-MRI better informs the surgical decision-making process and can lower the incidence of failed curative-intent pancreatic surgery. Consideration should be given to inclusion of EOB-MRI in the preoperative assessment of PDAC deemed potentially resectable on CECT.

## Supplementary Information

**Additional file 1: Table S1.** Diagnostic Imaging Interpretation Criteria.

## Data Availability

The datasets used and/or analyzed during the current study are available from the corresponding author on reasonable request.

## Abbreviations

CECT: Contrast-enhanced computed tomography
DWI: Diffusion-weighted imaging
DECT: Dual-energy CT scan
EOB-MRI: Gadoxetic acid-enhanced Magnetic Resonance Imaging
GEE: Generalized estimating eqs.
LM: Liver metastasis
NCCN: National Comprehensive Cancer Network
NPV: Negative predictive values
PDAC: Pancreatic ductal adenocarcinoma
PPV: Positive predictive value
SL: Staging laparoscopy

## References

[1] Siegel RL, Miller KD, Jemal A (2018). Cancer statistics, 2018. CA Cancer J Clin.

[2] McGuigan A, Kelly P, Turkington RC, Jones C, Coleman HG, McCain RS (2018). Pancreatic cancer: a review of clinical diagnosis, epidemiology, treatment and outcomes. World J Gastroenterol.

[3] Vincent A, Herman J, Schulick R, Hruban RH, Goggins M (2011). Pancreatic cancer. Lancet.

[4] Luchini C, Capelli P, Scarpa A (2016). Pancreatic ductal adenocarcinoma and its variants. Surg Pathol Clin.

[5] Somers I, Bipat S (2017). Contrast-enhanced CT in determining resectability in patients with pancreatic carcinoma: a meta-analysis of the positive predictive values of CT. Eur Radiol.

[6] Callery MP, Chang KJ, Fishman EK, Talamonti MS, William Traverso L, Linehan DC (2009). Pretreatment assessment of resectable and borderline resectable pancreatic cancer: expert consensus statement. Ann Surg Oncol.

[7] Liu X, Fu Y, Chen Q, Wu J, Gao W, Jiang K (2018). Predictors of distant metastasis on exploration in patients with potentially resectable pancreatic cancer. BMC Gastroenterol.

[8] Allen VB, Gurusamy KS, Takwoingi Y, Kalia A, Davidson BR (2016). Diagnostic accuracy of laparoscopy following computed tomography (CT) scanning for assessing the resectability with curative intent in pancreatic and periampullary cancer. Cochrane Database Syst Rev.

[9] Gemenetzis G, Groot VP, Blair AB, Ding D, Thakker SS, Fishman EK (2018). Incidence and risk factors for abdominal occult metastatic disease in patients with pancreatic adenocarcinoma. J Surg Oncol.

[10] Ito T, Sugiura T, Okamura Y, Yamamoto Y, Ashida R, Aramaki T (2017). The diagnostic advantage of EOB-MRI over CT in the detection of liver metastasis in patients with potentially resectable pancreatic cancer. Pancreatology.

[11] Chew C, O'Dwyer PJ (2016). The value of liver magnetic resonance imaging in patients with findings of resectable pancreatic cancer on computed tomography. Singap Med J.

[12] Jhaveri K, Cleary S, Audet P, Balaa F, Bhayana D, Burak K (2015). Consensus statements from a multidisciplinary expert panel on the utilization and application of a liver-specific MRI contrast agent (gadoxetic acid). AJR Am J Roentgenol.

[13] Jhaveri KS, Fischer SE, Hosseini-Nik H, Sreeharsha B, Menezes RJ, Gallinger S (2017). Prospective comparison of gadoxetic acid-enhanced liver MRI and contrast-enhanced CT with histopathological correlation for preoperative detection of colorectal liver metastases following chemotherapy and potential impact on surgical plan. HPB (Oxford).

[14] Kim HJ, Lee SS, Byun JH, Kim JC, Yu CS, Park SH (2015). Incremental value of liver MR imaging in patients with potentially curable colorectal hepatic metastasis detected at CT: a prospective comparison of diffusion-weighted imaging, gadoxetic acid-enhanced MR imaging, and a combination of both MR techniques. Radiology.

[15] Noda Y, Goshima S, Takai Y, Kawai N, Kawada H, Tanahashi Y (2020). Detection of pancreatic ductal adenocarcinoma and liver metastases: comparison of Gd-EOB-DTPA-enhanced MR imaging vs. extracellular contrast materials. Abdom Radiol (NY).

[16] Motosugi U, Ichikawa T, Morisaka H, Sou H, Muhi A, Kimura K (2011). Detection of pancreatic carcinoma and liver metastases with gadoxetic acid-enhanced MR imaging: comparison with contrast-enhanced multi-detector row CT. Radiology.

[17] Harris PA, Taylor R, Thielke R, Payne J, Gonzalez N, Conde JG (2009). Research electronic data capture (REDCap)--a metadata-driven methodology and workflow process for providing translational research informatics support. J Biomed Inform.

[18] Bartolozzi C, Cioni D, Donati F, Lencioni R (2001). Focal liver lesions: MR imaging-pathologic correlation. Eur Radiol.

[19] Horton KM, Bluemke DA, Hruban RH, Soyer P, Fishman EK (1999). CT and MR imaging of benign hepatic and biliary tumors. Radiographics.

[20] Sternberg MR, Hadgu A (2001). A GEE approach to estimating sensitivity and specificity and coverage properties of the confidence intervals. Stat Med.

[21] Core Team R (2018). R: a language and environment for statistical computing R Foundation for statistical computing, Vienna, Austria.

[22] National Comprehensive Cancer Network (2021). NCCN Clinical Practice Guidelines in Oncology (NCCN Guidelines): Pancreatic Adenocarcinoma (Version 2.2021).

[23] Alabousi M, McInnes MD, Salameh JP, Satkunasingham J, Kagoma YK, Ruo L (2021). MRI vs. CT for the detection of liver metastases in patients with pancreatic carcinoma: a comparative diagnostic test accuracy systematic review and meta-analysis. J Magn Reson Imaging.

[24] Berger-Kulemann V, Schima W, Baroud S, Koelblinger C, Kaczirek K, Gruenberger T (2012). Gadoxetic acid-enhanced 3.0 T MR imaging versus multidetector-row CT in the detection of colorectal metastases in fatty liver using intraoperative ultrasound and histopathology as a standard of reference. Eur J Surg Oncol.

[25] Tapper E, Kalb B, Martin DR, Kooby D, Adsay NV, Sarmiento JM (2011). Staging laparoscopy for proximal pancreatic cancer in a magnetic resonance imaging-driven practice: what's it worth?. HPB (Oxford).

[26] Ta R, O'Connor DB, Sulistijo A, Chung B, Conlon KC (2019). The role of staging laparoscopy in Resectable and borderline Resectable pancreatic Cancer: a systematic review and meta-analysis. Dig Surg.

[27] Conlon KC, Dougherty E, Klimstra DS, Coit DG, Turnbull AD, Brennan MF (1996). The value of minimal access surgery in the staging of patients with potentially resectable peripancreatic malignancy. Ann Surg.

[28] Morris S, Gurusamy KS, Sheringham J, Davidson BR (2015). Cost-effectiveness of diagnostic laparoscopy for assessing resectability in pancreatic and periampullary cancer. BMC Gastroenterol.

[29] Enestvedt CK, Mayo SC, Diggs BS, Mori M, Austin DA, Shipley DK (2008). Diagnostic laparoscopy for patients with potentially resectable pancreatic adenocarcinoma: is it cost-effective in the current era?. J Gastrointest Surg.

[30] Agrawal MD, Pinho DF, Kulkarni NM, Hahn PF, Guimaraes AR, Sahani DV (2014). Oncologic applications of dual-energy CT in the abdomen. Radiographics..

[31] Tsurusaki M, Sofue K, Hori M, Sasaki K, Ishii K, Murakami T (2021). Dual-energy computed tomography of the liver: uses in clinical practices and applications. Diagnostics (Basel).

[32] Vilgrain V, Esvan M, Ronot M, Caumont-Prim A, Aubé C, Chatellier G (2016). A meta-analysis of diffusion-weighted and gadoxetic acid-enhanced MR imaging for the detection of liver metastases. Eur Radiol.

