# Supplementary material for: Can preoperative liver MRI with gadoxetic acid help reduce open-close laparotomies for curative intent pancreatic cancer surgery?
Source: Cancer Imaging. 2021 Jun 30;21:45. doi: 10.1186/s40644-021-00416-4 (PMC8243548; doi:10.1186/s40644-021-00416-4)
Supplement: Supplementary file 1 — Additional file 1: Table S1. Diagnostic Imaging Interpretation Criteria. [file 40644_2021_416_MOESM1_ESM.docx]

**Table S1: Diagnostic Imaging Interpretation Criteria**

| **Imaging Modality** | **Metastasis** | **Benign** | **Indeterminate** |
| --- | --- | --- | --- |
| **CECT** | Ring or heterogeneous enhancement | Cyst: oval or round, well-defined, fluid attenuation, no contrast enhancement  Hemangioma: Peripheral nodular enhancement with centripetal fill-in on dynamic imaging, or  Focal fatty infiltration: hypodense geographic area, typical location, angular margins | Lesions not adhered to malignant or benign features, or  Hypodense lesions too small to characterize |
|  | | | |
| **EOB-MRI** | Ring or heterogeneous enhancement on dynamic imaging, and/or  Becoming hypointense on HBP, and/or  Hyperintense (but less than the signal of fluid) on T2-weighted FSE imaging, and/or  Restricted Diffusion with low ADC | Cyst: oval or round, well-defined, fluid intensity, no contrast enhancement or diffusion restriction  Hemangioma: Peripheral nodular enhancement with centripetal fill-in on dynamic imaging  Focal nodular hyperplasia: persistent lesion enhancement on HBP, no enhancement of central scar on HBP  Focal fatty infiltration: geographic area with fat intensity, typical location, angular margins | Lesions not adhered to malignant or benign features |

HBP = hepatobiliary phase
